# Supplementary material for: Modeling glioblastoma heterogeneity as a dynamic network of cell states
Source: Mol Syst Biol. 2021 Sep 16;17(9):e10105. doi: 10.15252/msb.202010105 (PMC8444284; doi:10.15252/msb.202010105)
Supplement: Supplementary file 6 — Source Data for Figure 5 [file MSB-17-e10105-s004.zip › Figure5A_sourcedata/GSEA_3017/nosigdb_stateB.GseaPreranked.1622795021725/FELDMAN_2020_G1.html]

Details for gene set FELDMAN\_2020\_G1[GSEA]

|  || Dataset | state43017 |
| Phenotype | NoPhenotypeAvailable |
| Upregulated in class | na\_pos |
| GeneSet | FELDMAN\_2020\_G1 |
| Enrichment Score (ES) | 0.67792946 |
| Normalized Enrichment Score (NES) | 3.989597 |
| Nominal p-value | 0.0 |
| FDR q-value | 0.0 |
| FWER p-Value | 0.0 |
Table: GSEA Results Summary

  

Fig 1: Enrichment plot: FELDMAN\_2020\_G1      
 Profile of the Running ES Score & Positions of GeneSet Members on the Rank Ordered List

  

| PROBE | GENE SYMBOL | GENE\_TITLE | RANK IN GENE LIST | RANK METRIC SCORE | RUNNING ES | CORE ENRICHMENT || 1 | HIST1H4C |  |  | 0 | 1.373 | 0.0234 | Yes |
| 2 | HIST1H1B |  |  | 1 | 1.272 | 0.0450 | Yes |
| 3 | TOP2A |  |  | 3 | 1.079 | 0.0619 | Yes |
| 4 | HIST1H1C |  |  | 4 | 1.041 | 0.0796 | Yes |
| 5 | HIST1H1E |  |  | 5 | 0.983 | 0.0963 | Yes |
| 6 | RRM2 |  |  | 7 | 0.875 | 0.1097 | Yes |
| 7 | UBE2C |  |  | 11 | 0.802 | 0.1187 | Yes |
| 8 | TPX2 |  |  | 12 | 0.801 | 0.1324 | Yes |
| 9 | NUSAP1 |  |  | 13 | 0.801 | 0.1460 | Yes |
| 10 | HMGB2 |  |  | 14 | 0.786 | 0.1594 | Yes |
| 11 | FBXO5 |  |  | 15 | 0.785 | 0.1728 | Yes |
| 12 | NCAPG |  |  | 16 | 0.778 | 0.1860 | Yes |
| 13 | SPC25 |  |  | 17 | 0.763 | 0.1990 | Yes |
| 14 | PRC1 |  |  | 18 | 0.756 | 0.2119 | Yes |
| 15 | CDCA2 |  |  | 19 | 0.751 | 0.2247 | Yes |
| 16 | ESCO2 |  |  | 20 | 0.742 | 0.2373 | Yes |
| 17 | GTSE1 |  |  | 22 | 0.732 | 0.2482 | Yes |
| 18 | KIF23 |  |  | 24 | 0.721 | 0.2590 | Yes |
| 19 | ASPM |  |  | 26 | 0.695 | 0.2693 | Yes |
| 20 | NDC80 |  |  | 27 | 0.695 | 0.2811 | Yes |
| 21 | ATAD2 |  |  | 28 | 0.685 | 0.2928 | Yes |
| 22 | KPNA2 |  |  | 33 | 0.676 | 0.2981 | Yes |
| 23 | CENPF |  |  | 34 | 0.670 | 0.3096 | Yes |
| 24 | KIF4A |  |  | 37 | 0.662 | 0.3178 | Yes |
| 25 | ANLN |  |  | 39 | 0.659 | 0.3275 | Yes |
| 26 | SMC4 |  |  | 41 | 0.655 | 0.3371 | Yes |
| 27 | CENPE |  |  | 43 | 0.654 | 0.3467 | Yes |
| 28 | NUF2 |  |  | 44 | 0.651 | 0.3578 | Yes |
| 29 | DLGAP5 |  |  | 46 | 0.647 | 0.3673 | Yes |
| 30 | DHFR |  |  | 49 | 0.638 | 0.3751 | Yes |
| 31 | CENPA |  |  | 51 | 0.636 | 0.3844 | Yes |
| 32 | CKAP2 |  |  | 53 | 0.628 | 0.3935 | Yes |
| 33 | CCNA2 |  |  | 54 | 0.626 | 0.4042 | Yes |
| 34 | AURKA |  |  | 55 | 0.617 | 0.4147 | Yes |
| 35 | CKAP2L |  |  | 59 | 0.611 | 0.4205 | Yes |
| 36 | CENPU |  |  | 60 | 0.605 | 0.4308 | Yes |
| 37 | CENPK |  |  | 62 | 0.603 | 0.4395 | Yes |
| 38 | TYMS |  |  | 63 | 0.601 | 0.4498 | Yes |
| 39 | CCDC34 |  |  | 65 | 0.595 | 0.4584 | Yes |
| 40 | IGFBP5 |  |  | 67 | 0.590 | 0.4669 | Yes |
| 41 | SMC2 |  |  | 68 | 0.586 | 0.4768 | Yes |
| 42 | CDK1 |  |  | 69 | 0.585 | 0.4868 | Yes |
| 43 | H2AFX |  |  | 71 | 0.576 | 0.4951 | Yes |
| 44 | CKS1B |  |  | 72 | 0.575 | 0.5049 | Yes |
| 45 | MAD2L1 |  |  | 77 | 0.569 | 0.5084 | Yes |
| 46 | PLK1 |  |  | 78 | 0.567 | 0.5181 | Yes |
| 47 | DBF4 |  |  | 79 | 0.567 | 0.5277 | Yes |
| 48 | CTGF |  |  | 81 | 0.564 | 0.5358 | Yes |
| 49 | CKS2 |  |  | 83 | 0.558 | 0.5438 | Yes |
| 50 | UBE2S |  |  | 87 | 0.555 | 0.5486 | Yes |
| 51 | AURKB |  |  | 89 | 0.548 | 0.5564 | Yes |
| 52 | CENPM |  |  | 92 | 0.544 | 0.5626 | Yes |
| 53 | CCNB1 |  |  | 93 | 0.544 | 0.5719 | Yes |
| 54 | PBK |  |  | 95 | 0.540 | 0.5795 | Yes |
| 55 | DEPDC1 |  |  | 97 | 0.536 | 0.5871 | Yes |
| 56 | TMEM106C |  |  | 100 | 0.523 | 0.5930 | Yes |
| 57 | RRM1 |  |  | 103 | 0.520 | 0.5988 | Yes |
| 58 | CMC2 |  |  | 105 | 0.516 | 0.6060 | Yes |
| 59 | CDKN3 |  |  | 108 | 0.513 | 0.6117 | Yes |
| 60 | UBE2T |  |  | 110 | 0.512 | 0.6189 | Yes |
| 61 | CDCA3 |  |  | 111 | 0.511 | 0.6276 | Yes |
| 62 | TUBB4B |  |  | 114 | 0.507 | 0.6331 | Yes |
| 63 | RAD21 |  |  | 115 | 0.506 | 0.6418 | Yes |
| 64 | BIRC5 |  |  | 116 | 0.504 | 0.6504 | Yes |
| 65 | CLSPN |  |  | 117 | 0.503 | 0.6589 | Yes |
| 66 | DTYMK |  |  | 118 | 0.503 | 0.6675 | Yes |
| 67 | DUT |  |  | 124 | 0.494 | 0.6682 | Yes |
| 68 | TMPO |  |  | 127 | 0.492 | 0.6735 | Yes |
| 69 | GMNN |  |  | 135 | 0.484 | 0.6711 | Yes |
| 70 | CYR61 |  |  | 145 | 0.465 | 0.6652 | Yes |
| 71 | ORC6 |  |  | 150 | 0.461 | 0.6670 | Yes |
| 72 | KIF22 |  |  | 152 | 0.459 | 0.6732 | Yes |
| 73 | KNSTRN |  |  | 155 | 0.456 | 0.6779 | Yes |
| 74 | TK1 |  |  | 167 | 0.444 | 0.6687 | No |
| 75 | MKI67 |  |  | 179 | 0.436 | 0.6593 | No |
| 76 | FEN1 |  |  | 180 | 0.435 | 0.6667 | No |
| 77 | CDC20 |  |  | 184 | 0.431 | 0.6694 | No |
| 78 | USP1 |  |  | 185 | 0.430 | 0.6767 | No |
| 79 | MIS18A |  |  | 196 | 0.421 | 0.6686 | No |
| 80 | ZWINT |  |  | 212 | 0.407 | 0.6526 | No |
| 81 | H2AFZ |  |  | 216 | 0.405 | 0.6549 | No |
| 82 | VRK1 |  |  | 238 | 0.388 | 0.6294 | No |
| 83 | PCNA |  |  | 242 | 0.386 | 0.6314 | No |
| 84 | HMGN2 |  |  | 243 | 0.386 | 0.6380 | No |
| 85 | CCNB2 |  |  | 250 | 0.379 | 0.6352 | No |
| 86 | CENPW |  |  | 267 | 0.366 | 0.6170 | No |
| 87 | ANP32E |  |  | 280 | 0.358 | 0.6047 | No |
| 88 | PSIP1 |  |  | 284 | 0.357 | 0.6062 | No |
| 89 | CALM2 |  |  | 290 | 0.354 | 0.6046 | No |
| 90 | CENPN |  |  | 309 | 0.343 | 0.5829 | No |
| 91 | DEK |  |  | 316 | 0.341 | 0.5796 | No |
| 92 | PHF19 |  |  | 319 | 0.339 | 0.5823 | No |
| 93 | DLG1 |  |  | 347 | 0.327 | 0.5465 | No |
| 94 | HAT1 |  |  | 352 | 0.324 | 0.5459 | No |
| 95 | LMNB1 |  |  | 363 | 0.321 | 0.5361 | No |
| 96 | PTTG1 |  |  | 413 | 0.302 | 0.4663 | No |
| 97 | C12orf75 |  |  | 433 | 0.296 | 0.4423 | No |
| 98 | MZT1 |  |  | 456 | 0.291 | 0.4136 | No |
| 99 | RNASEH2A |  |  | 478 | 0.285 | 0.3864 | No |
| 100 | NUCKS1 |  |  | 483 | 0.285 | 0.3851 | No |
| 101 | CENPH |  |  | 501 | 0.281 | 0.3639 | No |
| 102 | LSM3 |  |  | 522 | 0.277 | 0.3380 | No |
| 103 | DNMT1 |  |  | 523 | 0.277 | 0.3428 | No |
| 104 | GGH |  |  | 564 | 0.270 | 0.2862 | No |
| 105 | RPA3 |  |  | 584 | 0.267 | 0.2617 | No |
| 106 | EXOSC8 |  |  | 647 | 0.256 | 0.1713 | No |
Table: GSEA details [plain text format]

  

Fig 2: FELDMAN\_2020\_G1: Random ES distribution      
 Gene set null distribution of ES for **FELDMAN\_2020\_G1**
